# The Locus Coeruleus Noradrenaline System in Delirium

**DOI:** 10.3389/fnagi.2021.784356

**Published:** 2021-12-08

**Authors:** Niels Hansen, Alina Isabel Rediske

**Affiliations:** Department of Psychiatry and Psychotherapy, University Medical Center of Göttingen, Göttingen, Germany

**Keywords:** delirium, noradrenaline, locus coeruleus, cognition, attention

## Abstract

Delirium is a brain state involving severe brain dysfunction affecting cognitive and attentional capacities. Our opinion statement review aims to elucidate the relationship between abnormal arousal and locus coeruleus (LC) activity in cognitive dysfunction and inattention in delirium states. We propose (1) that enhanced noradrenaline release caused by altered arousal in hyperactive delirium states leads to increased noradrenergic transmission within the LC and subcortical and cortical brain regions including the prefrontal cortex and hippocampus, thus affecting how attention and cognition function. In hypoactive delirium states, however, we are presuming (2) that less arousal will cause the release of noradrenaline to diminish in the LC, followed by reduced noradrenergic transmission in cortical and subcortical brain areas concentrated within the prefrontal cortex and hippocampus, leading to deficient attention and cognitive processing. Studies addressing the measurement of noradrenaline and its derivatives in biomaterial probes regarding delirium are also covered in this article. In conclusion, the LC-NA system plays a crucial role in generating delirium. Yet there have been no large-scale studies investigating biomarkers of noradrenaline to help us draw conclusions for improving delirium’s diagnosis, treatment, and prognosis, and to better understand its pathogenesis.

## Functionality of the Locus Coeruleus in Delirium

Delirium is a transient human disease state based on a severe brain dysfunction often caused by an encephalopathy, as stated in a recent review by Wilson et al. (2020). The claim that brain dysfunction in delirium originates from an encephalopathy is a recent perspective that maintains that the encephalopathy’s pathophysiology can trigger delirium, but it fails to consider the latest clinical understanding of the terms encephalopathy and delirium. These two terms are clinically regarded as different disorders that are also handled by different clinical specialties. What both terms have in common is that they refer to disturbances in the brain’s function. However, delirium is clinical, usually more acute disease entity that requires a rapid clinical response, whereas the disease entity encephalopathy, as is its nature, tends to develop slowly and sometimes only reveals subtle abnormalities, as in hepatic encephalopathy (Wijdicks, 2016; Karanfilian et al., 2020) or drug-induced encephalopathy (Hansen, 2012) including antiepileptic-drug-induced encephalopathy (Hansen et al., 2010). Moreover, encephalopathies can also designate chronic brain states such as epileptic encephalopathies (for review, see Palmer et al., 2021) often sharing a genetic cause and encompassing a broad spectrum of developmental electroclinical syndromes characterized by subtle seizures and intellectual disabilities (like those caused by epileptic encephalopathies associated with KCNT1 mutations) (Ohba et al., 2015; Hansen et al., 2017). However, if one deconstructs the term encephalopathy to indicate its dysfunctionality affecting various brain functions, a manifest delirium could be a hint of an underlying chronic or acute encephalopathy. Several brain functions are impaired in delirium states such as arousal, cognition, and attention, leading to inattention, and deficits in orientation and cognitive dysfunction in distinct subdomains.

In this narrative review, we emphasize the role of a small brainstem nucleus called the locus coeruleus (LC) with its neurotransmitter noradrenaline (NA) in relation to delirium pathophysiology. The LC is known from rodent and human experiments to be innervated by fiber projections that involve both predominant excitatory neurotransmitters such as glutamate (Yang et al., 2021) and other mostly inhibitory neurotransmitters such as gamma aminobutyric acid (Hellsten et al., 2010; Wu et al., 2020) and galanin (Le Maître et al., 2013), so that ultimately, LC activity relies on a complex interplay between excitatory and inhibitory inputs to the LC.

The LC is located in the dorsal pons containing the main source of noradrenaline in the brain with a variety of LC fiber connections to cortical and subcortical brain regions (Schwartz and Luo, 2015), thus revealing its key function in the noradrenergic modulation of diverse brain functions such as attention and cognition. Arousal is a trigger for activating and regulating LC activity. Activated LC neurons can lead to NA’s release within the LC and its fiber LC terminals in various cognition-relevant brain regions (Feng et al., 2019) such as the prefrontal cortex and hippocampus. A high arousal state in humans might lead to high phasic activity of LC neurons that cause NA’s release in target regions such as the prefrontal cortex, whereas a low arousal state might cause tonic basal LC activity with less pronounced NA release in these brain regions (Berridge and Waterhouse, 2003). Noradrenergic neurons in the LC can be specifically activated by certain salient stimuli, as an optogenetic study showed in mice (Carter et al., 2010). We propose that stimuli in delirium that are not normally salient might alter its valency due to altered arousal states in delirium, meaning that normally non-salient stimuli can become salient in delirium and that salient stimuli might lose their saliency.

**FIGURE 1:**
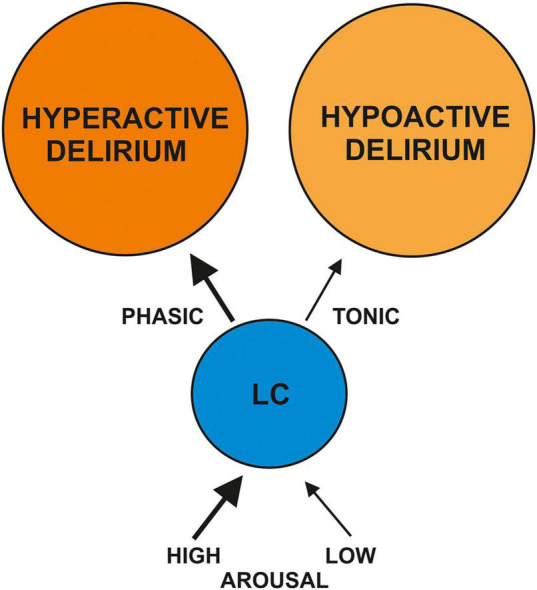
Delirium and the locus coeruleus (LC) noradrenaline system. This is a schematic hypothesis of how high or low arousal might induce more phasic vs. tonic LC activation. The altered LC activation might cause the hyper- or hypoactive forms of delirium via modulating the release of noradrenaline in different brain regions (bottom-up modulation); consequently, altered noradrenergic activity in brain regions might affect LC activity (top-down modulation).

Delirium consists of distinct phenotypic subtypes (Boettger and Breitbart, 2021) ranging from a hyperactive (encompassing agitation, aggressiveness, restlessness, or hallucinations as potential symptoms), a hypoactive (withdrawal, drowsiness, apathy, lethargy, or unresponsiveness as potential symptom spectrum), or mixed form. These different states of delirium might be triggered by altered LC-NA activity due to altered arousal states. An observational study confirmed that an abnormal arousal level (measured by the observational scale of arousal levels) can predict the occurrence of delirium (Tieges et al., 2013). This study demonstrates that abnormal arousal states are likely to be involved in generating delirium. We hypothesize that more NA release via high phasic LC activity in the LC and second, in fiber-projecting areas such as in the frontal cortex area and hippocampus might result in a hyperactive state of delirium, whereas a low NA release in the LC and its fiber projections targeting the frontal cortex area and hippocampus due to tonic LC activity is the consequence of low arousal activity that might be followed by a hypoactive form of delirium (Figure 1). Hyperactive delirium is more often associated with delusions or hallucinations than the hypoactive type (Boettger and Breitbart, 2021) suggesting another pathophysiology associated with greater dopamine release (according to the dopamine hypothesis for generating psychotic symptoms; for actual review, see Seeman, 2021). Another explanation is that the LC might also release dopamine in addition to NA, as a recent animal study showed that noradrenergic fiber terminals from LC might release dopamine in the hippocampus (Kempadoo et al., 2016). These observations could indicate that LC activation entailing a consecutive release of NA and dopamine in cognition-relevant brain regions is linked to the hyperactive form of delirium. A higher and uncontrolled NA release in the cortex and sensorimotor cortex might be responsible for deficits in attentional-executive functions and perceptual functions, as described in a recent review (Hansen, 2021). Agitation as feature of a hyperactive delirium is known to result from frontal lobe dysfunction involving a dysfunctional anterior cingulate and orbitofrontal cortex (recently reviewed by Carrarini et al., 2021). It is thus conceivable that a higher noradrenergic tonus results in a higher activity in these cortical brain regions that second might lead to upregulated ß-adrenergic receptors in these brain regions. These receptor anomalies might explain why weak stimuli could lead to higher NA activity (causing hypoactive delirium) and strong stimuli would cause global overactivation of noradrenergic receptors in the frontal cortex, corresponding to a hyperactive delirium state (Figure 1). Our hypothesis is supported by recent data that suggest that alpha2 receptor agonism by dexmedetomidine reduces the occurrence of delirium in surgery patients (Su et al., 2016; Deiner et al., 2017; Turan et al., 2020) and even prevents delirium in patients (Skrobik et al., 2018). An alpha2 receptor agonism might reduce noradrenaline release in the LC, according to evidence from rodents in a study by Callado and Stamford (1999). Thus, alpha2 receptor agonism by dexmedetomidine in the long-term should result in lower levels of noradrenaline release, favoring the inhibition of hyperactive delirium development and impeding the conditions for maintaining a delirium.

There is evidence that neurons from the LC degenerate in AD (Olivieri et al., 2019) and other neurodegenerative diseases such as dementia with Lewy bodies or Parkinson’s disease dementia (Hansen, 2021). Pre-existing neurodegenerative disease might facilitate the development of delirium (Davis et al., 2015; Zipser et al., 2019; Wilson et al., 2020) due to the brain’s increased greater vulnerability when reacting to stress. Degenerated LC neurons might predispose the brain to exhibit abnormal arousal reactions due to altered NA content in the LC. That might explain the higher risk of delirium generation in patients with a degenerated LC-NA system, such as in Alzheimer’s dementia.

## Locus Coeruleus Activity Causes Inattention in Delirium

Locus coeruleus activity is tightly linked to attentional processes. High LC activity due to strong arousal drives attentional prioritization within objects (O’Bryan and Scolari, 2021), selective attention (Dahl et al., 2020), and focused attention (Aston-Jones et al., 1999). In contrast, low LC activity might be relevant for high behavioral flexibility and a state in which attentional functions require a low basal state to prepare for increasing activation in situations demanding a specific task performance (Aston-Jones et al., 1999). If the arousal system is abnormal in a delirium state, LC activity is consecutively dysfunctional, and attentional bias might result, so that attention cannot be prioritized, focused, or selective. Moreover, cognitive flexibility is also lost through the change in arousal. The LC’s role in the attention network is well understood, and several attentional disorders are caused by a dysfunctioning LC (Keehn et al., 2021; Kershner, 2021). In addition, the balance between tonic and phasic LC activity might be dysregulated in delirium states, as a synergistic interplay between the tonic and phasic LC mode is relevant for optimal attention performance (Howells et al., 2012). Another factor is that the processing of salient information can be dysregulated in delirium states. Attentional working memory is disturbed by dysfunctional LC-NA activity due to projections from the LC to the prefrontal cortex, resulting in poorer working-memory performance via a noradrenergic bottom-up modulation. Such a poor working-memory performance itself can lead to less attentional control (Unsworth and Robison, 2017) via a noradrenergic top-down modulation. We propose that an abnormal arousal in delirium leads to altered LC activity and attentional dysfunction that mainly determine the delirium phenotype that appears (hyperactive vs. hypoactive). The LC-NA system is very relevant, as modeling investigations have suggested its stronger relevance (than that of other neuromodulatory systems such as the serotonergic, cholinergic, and dopaminergic) (Plini et al., 2021). Another aspect to be aware of is the upregulation of ß-adrenoreceptors in the brain if the noradrenergic tonus is continuously elevated, as it is in states of hyperactive delirium. The upregulation of ß-adrenoreceptors needs substantial time to develop, which would rule out the acute effects of promoting delirium. However, if a delirium is not stopped rapidly, the ß-adrenoreceptor distribution could lead to various effects in attentional and cognitive function that might be relevant factors in maintaining or facilitating delirium states.

## Locus Coeruleus-Dependent Modulation of Cognition in Delirium

Various animal and human studies have shown that noradrenaline from the LC is involved in cognition (Berridge and Waterhouse, 2003; Sara, 2009; Sara and Bouret, 2012; Borodovitsyna et al., 2017; Jacobs et al., 2020). In particular, the task-specific modulation of LC activity required for specific cognitive functions might be disrupted in delirium via a bottom-up or top-down noradrenergic modulation of brain regions, such as the interaction between the prefrontal cortex or hippocampus and LC. The LC-NA system regulates hippocampal synaptic plasticity and different stages of memory processing in rodents and humans (Hansen and Manahan-Vaughan, 2015; Hansen, 2017; Jacobs et al., 2020). Recent evidence suggests (Grella et al., 2021) that the recruitment of new neurons in particular is dependent on ß-adrenoreceptor-dependent process regulated by the LC-NA system in order to enhance hippocampal informational processing of contextual information. This evidence makes us suspect that information entering the hippocampal system cannot be processed adequately in delirium states due to the LC-NA system’s failure. However, it is not just the LC-NA system that might be impaired—also target brain regions like the hippocampus or prefrontal cortex might be functionally disrupted in delirium through an acute encephalopathy attributable to various factors (infectious, autoimmune, starting neurodegeneration, cancer, etc.).

## The Locus Coeruleus Noradrenaline System’s Potential Role in Delirium

Many different brain functions and the LC-NA system’s involvement in delirium patients are being investigated. We will review measurements of NA and its derivatives in delirium in order to verify the LC-NA system’s obvious involvement in generating delirium. A recent study of hip-fracture patients showed that cerebrospinal fluid (CSF) adrenaline levels are lower in delirious patients than in those without delirium (Henjum et al., 2021). That finding is surprising, as more pronounced LC activation would be expected in hyperactive delirium, resulting in stronger NA release. Nevertheless, those findings suggest that the release of NA and NA derivatives might also be inhibited in delirium. The potential explanation for this observation is a loss of neurons or dysfunctional LC activation in conjunction with autoinhibited alpha2-adrenoreceptor activation. Another possible explanation is that arousal is probably high in hyperactive delirium, but might be too low to fulfill attentional and cognitive demands. The finetuning of LC activity might be severely compromised by the delirium state. The fact that the noradrenergic LC system plays a key role in delirium was supported by a study showing that the serum level of NA in an intermediate care unit at admission was associated with the risk of developing delirium there (Yasuda et al., 2020). However, these study results must be interpreted with caution as the noradrenaline in peripheral blood does not necessarily reflect central noradrenergic activity, although it might be interrelated in several disease states in which the blood–brain barrier is disturbed, as in delirium states.

Another indirect hint was revealed in a study that addressed amino acids in delirium patients. The authors reported an increased ratio between phenylalanine and the large neutral amino acids, suggesting greater synthesis of NA and dopamine in delirium (van der Mast et al., 2000). In delirium related to alcohol, e.g., alcohol withdrawal syndrome, NA was clearly elevated in the CSF in the acute stage, dropping later to normal levels in a study by Hawley et al. (1981) with 19 patients. An older study confirmed those findings in NA and noradrenaline derivatives in urine, 3-methoxy-4-hydroxyphenylglycol as a metabolite of NA degradation in the CSF and dopamine beta-hydroxylase in serum in alcohol withdrawal syndrome (Athen et al., 1977). The effect of increased NA led to neuronal injury and delirium in an animal model (Bhardwaj et al., 1990). Another reason why NA might be elevated in delirium is that sympathetic activity rises in delirium (hyperactive delirium) and that rise itself leads to increased circulation of NA derivatives in the blood. These examples show that NA and its derivatives in blood and the CSF might be altered in specific types of delirium through an overactivated or inhibited LC system following changes in arousal.

## Former and Current Treatment Strategies for Delirium Based on the Noradrenergic Locus Coeruleus System

Our depicted knowledge of the potential involvement of the LC-NA system in delirium and diverse studies (Su et al., 2016; Deiner et al., 2017; Subramaniam et al., 2019; Turan et al., 2020) underly the usefulness of alpha2 receptor agonists such as dexmedetomidine in delirium. Dexmedetomidine might be helpful to dampen an overall excitation by a high noradrenergic tonus resulting in a hyperactive delirium. Earlier and the latest studies indicate that dexmedetomidine application is beneficial in patients to prevent delirium (Su et al., 2016; Deiner et al., 2017), but also in already developed delirium for reducing symptoms (Liu et al., 2017). A beta-adrenoreceptor blocker proved to be effective in delirium treatment in 72 patients with alcohol-induced delirium (Golodets and Govoroin, 1983). Dexmedetomidine does not worsen sleep architecture if applied nocturnally in a small dosage in critically ill patients (Skrobik et al., 2018). In their investigation, the nocturnal application of dexmedetomidine also helped reduce the incidence of delirium in those patients. These studies demonstrate that the LC-NA system is a relevant drug target for delirium therapy. More studies should be conducted with large patient groups and distinguishing between delirium forms, and investigating a variety of drugs modulating the LC-NA system in diverse application settings (oral versus intravenous) and time intervals (short-term, interval vs. long-term treatment) to delineate the usefulness of drugs that modulate the LC-NA system in delirium.

## Synopsis

We assume that the LC-NA is involved in inducing delirium and its symptomatic spectrum. These conclusions are derived from incorporating the LC-NA system’s essential integrity in guaranteeing optimal attention and cognition. Cognitive dysfunction and inattention are caused by a dysregulation of the LC-NA system. Investigations in delirium patients suggest increased activity of the sympathetic system leading to higher NA tonus. However, as delirium patients suffering a bone fracture also present decreased NA and its derivatives, it is more likely that unusual alterations, that is, a sudden rise or fall in NA levels in the blood and CSF might be what affects the cognitive and attentional functions apparent in delirium patients.

## Conclusion

Our reflections emphasize the importance of the LC-NA system in generating delirium. Nevertheless, its diagnostic and therapeutic implications remain unclear. More large-scale and intensive research endeavors are needed to clarify the LC-NA system’s multifaceted role. Arousal-driven delirium is a brain state that enables us to study the LC-NA system during an upregulated state that impairs NA-dependent functions such as cognition and attention.

## Author Contributions

NH wrote and conceptualized the manuscript. AIR revised the manuscript for important intellectual content. Both authors contributed to the article and approved the submitted version.

## Conflict of Interest

The authors declare that the research was conducted in the absence of any commercial or financial relationships that could be construed as a potential conflict of interest.

## Publisher’s Note

All claims expressed in this article are solely those of the authors and do not necessarily represent those of their affiliated organizations, or those of the publisher, the editors and the reviewers. Any product that may be evaluated in this article, or claim that may be made by its manufacturer, is not guaranteed or endorsed by the publisher.
